# CASE REPORT: GUILLAIN-BARRE SYNDROME, AN UNUSUAL PRESENTATION

**DOI:** 10.51894/001c.123042

**Published:** 2024-08-30

**Authors:** Ania Pathak

**Affiliations:** 1 College of Osteopathic Medicine Michigan State University

## 62

### INTRODUCTION

Acute Inflammatory Demyelinating Polyradiculoneuropathy (AIDP), historically often referred to as Guillain-Barre syndrome, is an immune-mediated demyelination of nerve cells of unknown etiology, leading to symmetrical limb weakness and areflexia. Typical presentation includes a progressive ascending paralysis 2-3 weeks after diarrhea or respiratory illness. Classic presentation begins with weakness and tingling of the legs that progresses to muscle weakness and can ultimately progress to paralysis and death. Early recognition of AIDP across atypical and complex clinical manifestations would allow for timely confirmatory diagnosis and treatment administration, leading to improved clinical outcomes. This case report aims to add evidence for keeping AIDP as a differential diagnosis when sensory and strength deficits begin in the upper limbs, even in light of cervical stenosis findings.

### CASE DESCRIPTION

A woman in her 60s with a recent history of diarrhea presented to a local hospital emergency room with concerns for weakness. She had been developing progressive bilateral hand weakness for several days, and recently began to have a hard time standing and walking. Only when she became unable to get herself to the restroom did she come to the hospital. MRI of the head and neck were performed, revealing evidence of cervical stenosis, but no evidence of stroke. Initially, the bimanual loss of function was attributed to stenosis of the cervical spine. Nerve conduction studies were performed and revealed AIDP. She was treated with 5 days of IVIg, and transferred to rehabilitative medicine for continued care in regaining function.

### CONCLUSION

It is important to consider AIDP in patients who present atypically. Namely, symptoms of upper limb involvement first as opposed to an ascending paralysis beginning in the legs, as well as diagnostic evidence that supports cervical stenosis should not rule out AIDP, especially in the context of a known precipitating factor, history of recent diarrhea.

